# Computation of Propagating and Non-Propagating Lamb-Like Wave in a Functionally Graded Piezoelectric Spherical Curved Plate by an Orthogonal Function Technique

**DOI:** 10.3390/ma11122363

**Published:** 2018-11-23

**Authors:** Xiaoming Zhang, Shunli Liang, Xiaoming Han, Zhi Li

**Affiliations:** School of Mechanical and Power Engineering, Henan Polytechnic University, Jiaozuo 454003, China; zxmworld11@hpu.edu.cn (X.Z.); 18848957628@163.com (S.L.); jixielizhi@126.com (Z.L.)

**Keywords:** non-propagating wave, functionally graded piezoelectric material, orthogonal function technique, dispersion, displacement distribution

## Abstract

Non-propagating waves have great potential for crack evaluation, but it is difficult to obtain the complex solutions of the transcendental dispersion equation corresponding to the non-propagating wave. This paper presents an analytical approach based on the orthogonal function technique to investigate non-propagating Lamb-like waves in a functionally graded piezoelectric spherical curved plate. The presented approach can transform the set of partial differential equations for the acoustic waves into an eigenvalue problem that can give the generally complex wave numbers and the field profiles. A comparison of the obtained results with the well-known ones in plates is provided. The obtained solutions of the dispersion equation are shown graphically in three dimensional frequency-complex wave number space, which aids in understanding the properties of non-propagating waves better. The properties of the guided wave, including real, purely imaginary, and complex branches in various functionally graded piezoelectric spherical curved plates, are studied. The effects of material piezoelectricity, graded fields, and mechanical and electrical boundary conditions on the dispersion characteristics, are illustrated. The amplitude distributions of displacement and electric potential are also discussed, to analyze the specificities of non-propagating waves.

## 1. Introduction

Guided ultrasonic waves (GUW) are widely used as a tool for various problems in structural health monitoring and nondestructive evaluation. Lamb or Lamb-like waves are of great interest for nondestructive testing of the structures, due to their long propagation distances and their potential for interacting with defects [1,2]. However, some difficulties can arise in the recognition and in the interpretation of some parts of signals that are caused by the large number of practical interaction problems between defects and guided waves. If the defects are near the edge of a structure, the interaction would be much more sophisticated, since the wave field near the defect or the edge is transformed, and it represents a diverse superposition of propagating and non-propagating (evanescent) modes [3,4]. Non-propagating waves would play an important role in the reconstruction of defect shapes. These waves can be practically detected in the close vicinity of discontinuities. The error would be generated when processing the guided wave signal without considering non-propagating modes, which leads to identification errors regarding the shapes and sizes of defects. Therefore, it is necessary to have a deep understanding of the non-propagating waves. However, the research on non-propagating waves in waveguides is limited, especially for demanding cases such as those involving composite materials or curved structures.

In recent years, functionally graded piezoelectric materials (FGPMs) have received considerable attention, with widespread use in acoustic electronic devices, measuring instruments, and vibration control devices [5]. FGPM can improve the important physical properties of coupling between electrical and mechanical properties, and interface problems, because of the intrinsic advantages of functionally graded material (FGMs), thus increasing the reliability and lifespan of the modern piezoelectric structures [6]. Many applications are tightly related to the wave propagation of FGPM. Demands from the nondestructive evaluation and ultrasonic technology fields make the study of wave propagation in FGPM structures a topic of practical importance. There are many computational models and methods that are available in literature to investigate different waves propagating in various FGPM structures, including the special function method [7,8], the power series method [9], the Legendre polynomial method [10,11], the inhomogeneous layer element method [12], the Wentzel–Kramers–Brillouin [13,14], the spectral element method [15], and the Peano-series expansion method [16]. The above investigations are confined to propagating waves. Indeed, it is difficult to gain the complete root of the transcendental dispersion equation relating the wave number to the frequency. The real roots corresponding the propagating waves can be solved easily, but the complex roots corresponding the non-propagating waves are difficult to obtain.

Unlike propagating wave modes, non-propagating wave modes with non-real wave numbers decay with propagating distance. Thus, they are usually referred to as evanescent or non-propagating waves. Many studies have shown the importance of complex waves describing the interaction phenomena at the vicinity of the defects. Lyon [17] obtained the purely imaginary wave number solutions for an elastic plate as early as 1955. Later, Mindlin [18] presented the complete spectrum, including real, imaginary, and complex branches. Using the numerical spectral method, Pagneux and Maurel [19] determined the complex Lamb wave spectrum; Quintanilla et al. [20] computed the full dispersion solutions for guided waves in plate and cylinder structures with generally anisotropic media. Chen et al. [21] investigated the shear horizontal (SH) waves theoretically with purely imaginary wave numbers in a piezoelectric plate. Using the boundary element method, Daros [22] investigated the SH waves in a class of inhomogeneous anisotropic media, and presented the stress intensity factors-frequency curves for exponential inhomogeneous solid in non-propagating frequency range. Yan and Yuan [23] used a semi-analytical approach to investigate wave mode conversion from the SH evanescent wave into the propagating wave. Using spectral methods, Dubuc et al. [24] studied propagating and non-propagating guided waves in a nonuniformly stressed plate. These studies on non-propagating waves heavily focused on simple materials and structures, such as isotropic material and flat plate structures. More recently, non-propagating waves in functionally graded piezoelectric cylindrical structures with sectorial cross-sections, and in functionally graded piezoelectric-piezomagnetic plates, were investigated by Zhang et al. [25] and Zhang et al. [26] without considering the different mechanical and electrical boundary conditions. So far, the spherical curved structures have been seldom studied, which have considerable difficulty in obtaining the complete dispersion solutions. To the best of the authors’ knowledge, the non-propagating waves in FGPM spherical curved structures, with different mechanical and electrical boundary conditions, have not been studied before.

The conventional approaches (root-finding routines or finite element simulations) require a tedious iterative search procedure or a far greater coding effort to find complex roots. In this paper, we present a method that is based on the orthogonal function technique, to determine the complex dispersion solution, and to study the characteristics of non-propagating guided waves in a FGPM spherical curved plate. The presented method can convert the complicated acoustic wave equations into a classical eigenvalue problem, which can directly determine complex wave numbers for a specified real frequency. The complete dispersion curves are plotted in three-dimensional (3D) frequency-complex wave number space. Two known cases are given to validate the correctness of the presented method. The influences of piezoelectricity, graded fields, and mechanical and electrical boundary conditions on the dispersion curves are illustrated. The amplitude distributions of the displacement and electric potential are also discussed in detail.

## 2. Statement of the Problem and Basic Equations

**Figure 1:**
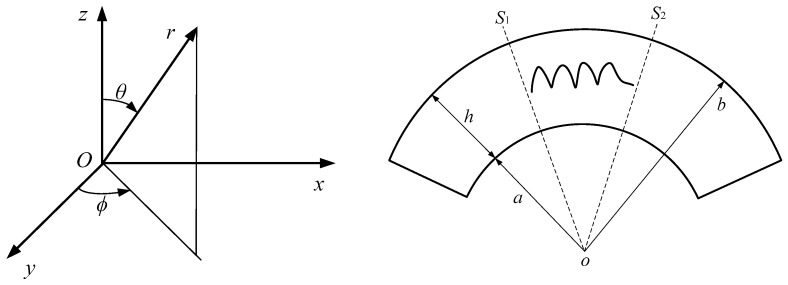
The model of the spherical curved plate.

Consider a FGPM spherical curved plate with material properties varying gradually in the *r* direction. The spherical coordinate system (r,θ,φ) is used to describe the wave propagation problem, as shown in Figure 1. Let *a* and *b* respectively denote the inner and outer radius, *h* the thickness, and the radius–thickness ratio η=b/h.

The governing field equations and the constitutive relations can be expressed as Equations (1) and (2), respectively [9]:

(1) $$\left\{ \begin{array}{l} \frac{\partial T_{rr}}{\partial r}+\frac{1}{r}\frac{\partial T_{r\theta }}{\partial \theta }+\frac{1}{r\sin\theta }\frac{\partial T_{r\varphi }}{\partial \varphi }+\frac{2T_{rr}+T_{r\theta }\cot\theta -T_{\theta \theta }-T_{\varphi \varphi }}{r}=\rho \frac{\partial^{2}u_{r}}{\partial t^{2}}\\ \frac{\partial T_{r\theta }}{\partial r}+\frac{1}{r}\frac{\partial T_{\theta \theta }}{\partial \theta }+\frac{1}{r\sin\theta }\frac{\partial T_{\theta \varphi }}{\partial \varphi }+\frac{3T_{r\theta }+\cot\theta \left(T_{\theta \theta }-T_{\varphi \varphi }\right)}{r}=\rho \frac{\partial^{2}u_{\theta }}{\partial t^{2}}\\ \frac{\partial T_{r\varphi }}{\partial r}+\frac{1}{r}\frac{\partial T_{\theta \varphi }}{\partial \theta }+\frac{1}{r\sin\theta }\frac{\partial T_{\varphi \varphi }}{\partial \varphi }+\frac{3T_{r\varphi }+2T_{\theta \varphi }\cot\theta }{r}=\rho \frac{\partial^{2}u_{\varphi }}{\partial t^{2}}\\ \frac{\partial D_{r}}{\partial r}+\frac{1}{r}\frac{\partial D_{\theta }}{\partial \theta }+\frac{1}{r\sin\theta }\frac{\partial D_{\varphi }}{\partial \varphi }+\frac{2D_{r}+D_{\theta }\cot\theta }{r}=0 \end{array}\right.\;$$

(2a) $$\left\{\begin{array}{c} T_{\theta \theta }\\ T_{\phi \phi }\\ T_{rr}\\ T_{r\phi }\\ T_{r\theta }\\ T_{\theta \phi } \end{array}\right\}=\left[\begin{array}{cccccc} C_{11} & C_{12} & C_{13} & C_{14} & C_{15} & C_{16}\\ & C_{22} & C_{23} & C_{24} & C_{25} & C_{26}\\ & & C_{33} & C_{34} & C_{35} & C_{36}\\ & & & C_{44} & C_{45} & C_{46}\\ & symmetry & & & C_{55} & C_{56}\\ & & & & & C_{66} \end{array}\right]\left\{\begin{array}{c} \varepsilon_{\theta \theta }\\ \varepsilon_{\phi \phi }\\ \varepsilon_{rr}\\ 2\varepsilon_{r\phi }\\ 2\varepsilon_{r\theta }\\ 2\varepsilon_{\theta \phi } \end{array}\right\}-\left[\begin{array}{ccc} e_{11} & e_{21} & e_{31}\\ e_{12} & e_{22} & e_{32}\\ e_{13} & e_{23} & e_{33}\\ e_{14} & e_{24} & e_{34}\\ e_{15} & e_{25} & e_{35}\\ e_{16} & e_{26} & e_{36} \end{array}\right]\left\{\begin{array}{c} E_{\theta }\\ E_{\phi }\\ E_{r} \end{array}\right\}\;$$

(2b) $$\left\{\begin{array}{c} D_{\theta }\\ D_{\phi }\\ D_{r} \end{array}\right\}=\left[\begin{array}{cccccc} e_{11} & e_{12} & e_{13} & e_{14} & e_{15} & e_{16}\\ e_{21} & e_{22} & e_{23} & e_{24} & e_{25} & e_{26}\\ e_{31} & e_{32} & e_{33} & e_{34} & e_{35} & e_{36} \end{array}\right]\left\{\begin{array}{c} \varepsilon_{\theta \theta }\\ \varepsilon_{\phi \phi }\\ \varepsilon_{rr}\\ 2\varepsilon_{r\phi }\\ 2\varepsilon_{r\theta }\\ 2\varepsilon_{\theta \phi } \end{array}\right\}+\left[\begin{array}{ccc} \in_{11} & \in_{12} & \in_{13}\\ & \in_{22} & \in_{23}\\ symmetry & & \in_{33} \end{array}\right]\left\{\begin{array}{c} E_{\theta }\\ E_{\phi }\\ E_{r} \end{array}\right\}\;$$

The generalized geometric relations under a spherical coordinate system are as the following Equation (3):

(3) $$\varepsilon_{rr}=\frac{\partial u_{r}}{\partial r},\;\varepsilon_{\theta \theta }=\frac{1}{r}\frac{\partial u_{\theta }}{\partial \theta }+\frac{u_{r}}{r},\;\varepsilon_{\varphi \varphi }=\frac{1}{r\sin\theta }\frac{\partial u_{\varphi }}{\partial \varphi }+\frac{u_{r}}{r}+\frac{\cot\theta }{r}u_{\theta },\;\;\varepsilon_{r\theta }=\frac{1}{2}\left(\frac{1}{r}\frac{\partial u_{r}}{\partial \theta }+\frac{\partial u_{\theta }}{\partial r}-\frac{u_{\theta }}{r}\right),\;\varepsilon_{r\varphi }=\frac{1}{2r}\left(\frac{1}{\sin\theta }\frac{\partial u_{r}}{\partial \varphi }-u_{\varphi }\right)+\frac{1}{2}\frac{\partial u_{\varphi }}{\partial r},\;\;\varepsilon_{\theta \varphi }=\frac{1}{2r}\left(\frac{1}{\sin\theta }\frac{\partial u_{\theta }}{\partial \varphi }+\frac{\partial u_{\varphi }}{\partial \theta }-u_{\varphi }\cot\theta \right),\;E_{r}=-\frac{\partial \Phi }{\partial r},\;E_{\theta }=-\frac{1}{r}\frac{\partial \Phi }{\partial \theta },\;E_{\varphi }=-\frac{1}{r\sin\theta }\frac{\partial \Phi }{\partial \varphi }\;$$

In the above three equations, $T_{ij}$ and $D_{i}$ respectively denote the stress and electric displacement. $C_{ij},\;e_{ij}$ and $\in_{ij}$ denote the elastic, piezoelectric, and dielectric parameters of the FGPM, respectively. $\varepsilon_{ij}\text{​}$ and $E_{i}$ denote the strain and the electric field, respectively. *u_i_* (*i* = *r*, *θ*, *φ*) denotes the mechanical displacement component in the *i*th direction. Φ is electric potential, and *ρ* is mass density.

The material parameters vary gradually in *r* direction; thus, they are the functions of *r* and can be fitted into:

(4) $$f(r)=f^{\left(l\right)}{\left(r/h\right)}^{l},l=0,1,2\ldots ,L\;$$

where *l* is the order number, *f* represents the material parameters, namely *C*, *e*, ∈ and *ρ*. *f*^(*l*)^ are the coefficients that are determined to fit the polynomials into the initial material constants. For homogeneous material, *f*(*r*) = *f* ^(0)^ and *f* ^(*l*)^ are 0 when *l* > 0.

Different boundary conditions are considered as follows. For the traction-free boundary condition, it requires that $T_{rr}|{}_{r=a,b}=0$, $T_{r\theta }|{}_{r=a,b}=0$, $T_{r\phi }|{}_{r=a,b}=0$. For the mechanical fixed boundary condition, $u_{i}|{}_{r=a,b}=0$. For the electric open circuit, $D_{r}|{}_{r=a,b}=0$, and for the electric closed circuit, $\Phi |{}_{r=a,b}=0$. Taking the traction-free and electricity open-circuit case as an example, we introduce a rectangular window function X(r) to satisfy the material parameters depending on the position, which can be expressed as:

(5) $$f(r)=f\cdot X(r),\;X(r)=\left\{ \begin{array}{l} \begin{array}{cc} 1, & a\leq r\leq b \end{array}\\ \begin{array}{cc} 0, & elsewhere \end{array} \end{array}\right.\;$$

The derivative of the rectangular window function is δ(r−a)−δ(r−b), with *δ* being a step function. This treatment can automatically introduce the boundary conditions into the wave propagation equations [27,28].

For a free harmonic wave propagating in the circumferential direction of a piezoelectric spherical waveguide, the mechanical displacement and electric potential can be expressed as:

(6) $$u_{i}=\exp(ikb\phi -i\omega t)U_{i}(r),\;\Phi =\exp(ikb\phi -i\omega t)Y(r)\;$$

where *U_i_* and *Y* represent the vibration amplitude in the *i*th (*i* = *r*, *θ*, *φ*) direction and the amplitude of the electric potential, respectively. *k* is wave number, ω is the angular frequency, and *i* is the imaginary number.

Substituting Equations (3)–(6) into Equation (2) with following substitution into Equation (1), we can obtain the governing differential equations in terms of the displacement and electric potential components. Here, the case of an orthotropic FGPM spherical curved plate with polarization in the thickness direction is given:

(7a) $$\begin{array}{l} \frac{1}{h^{l}}\{r^{l+2}\left(C_{33}^{\left(l\right)}U^{\prime \prime }+e_{33}^{\left(l\right)}Y^{\prime \prime }\right)+r^{l+1}[\left(l+2\right)C_{33}^{\left(l\right)}U^{\prime }+ikb\left(C_{23}^{\left(l\right)}+C_{44}^{\left(l\right)}\right)W^{\prime }+(\left(l+2\right)e_{33}^{\left(l\right)}-e_{31}^{\left(l\right)}-e_{32}^{\left(l\right)})Y^{\prime }]+r^{l}[\left(l+1\right)\left(C_{13}^{\left(l\right)}+C_{23}^{\left(l\right)}\right)U\\ -\left(C_{11}^{\left(l\right)}+C_{22}^{\left(l\right)}+2C_{12}^{\left(l\right)}+k^{2}b^{2}C_{44}^{\left(l\right)}\right)U+ikb\left(\left(l+1\right)C_{23}^{\left(l\right)}-C_{12}^{\left(l\right)}-C_{22}^{\left(l\right)}\right)W-C_{44}^{\left(l\right)}W-k^{2}b^{2}e_{24}^{\left(l\right)}Y]\}X\left(r\right)\\ +\left(\delta (r-a)-\delta (r-b)\right)\frac{1}{h^{l}}\{r^{l+2}\left(C_{33}^{\left(l\right)}U^{\prime }+e_{33}^{\left(l\right)}Y^{\prime }\right)+r^{l+1}\left[\left(C_{13}^{\left(l\right)}+C_{23}^{\left(l\right)}\right)U+ikbC_{23}^{\left(l\right)}W\right]\}=-\frac{\rho^{\left(l\right)}r^{l+2}\omega^{2}}{h^{l}}UX\left(r\right) \end{array}$$

(7b) $$\begin{array}{l} \frac{1}{h^{l}}\{r^{l+2}C_{55}^{\left(l\right)}V^{\prime \prime }+r^{l+1}\left(l+2\right)C_{55}^{\left(l\right)}V^{\prime }-r^{l}(\left(l+2\right)C_{55}^{\left(l\right)}+C_{12}^{\left(l\right)}+k^{2}b^{2}C_{66}^{\left(l\right)})V\}X\left(r\right)+\left(\delta (r-a)-\delta (r-b)\right)\frac{1}{h^{l}}\cdot \\ \{r^{l+2}C_{55}^{\left(l\right)}V^{\prime }-r^{l+1}C_{55}^{\left(l\right)}V\}=-\frac{\rho^{\left(l\right)}r^{l+2}\omega^{2}}{h^{l}}VX\left(r\right) \end{array}$$

(7c) $$\begin{array}{l} \frac{1}{h^{l}}\{r^{l+2}C_{44}^{\left(l\right)}W^{\prime \prime }+r^{l+1}[\left(ikbC_{23}^{\left(l\right)}+ikbC_{44}^{\left(l\right)}\right)U^{\prime }+\left(l+2\right)C_{44}^{\left(l\right)}W^{\prime }+ikb\left(e_{32}^{\left(l\right)}+e_{24}^{\left(l\right)}\right)Y^{\prime }]+r^{l}[ikb\left(\left(l+2\right)C_{44}^{\left(l\right)}+C_{12}^{\left(l\right)}+C_{22}^{\left(l\right)}\right)U\\ +\left(C_{66}^{\left(l\right)}-\left(l+2\right)C_{44}^{\left(l\right)}-k^{2}b^{2}C_{22}^{\left(l\right)}\right)W+ikb\left(l+2\right)e_{24}^{\left(l\right)}Y]\}X\left(r\right)+\left(\delta (r-a)-\delta (r-b)\right)\frac{1}{h^{l}}\{r^{l+2}C_{44}^{\left(l\right)}W^{\prime }\\ +r^{l+1}\left(ikbC_{44}^{\left(l\right)}U-C_{44}^{\left(l\right)}W+ikbe_{24}^{\left(l\right)}Y\right)\}=-\frac{\rho^{\left(l\right)}r^{l+2}\omega^{2}}{h^{l}}WX\left(r\right) \end{array}$$

(7d) $$\begin{array}{l} \frac{1}{h^{l}}\{r^{l+2}\left(e_{33}^{\left(l\right)}U^{\prime \prime }-\in_{33}^{\left(l\right)}Y^{\prime \prime }\right)+r^{l+1}[\left(e_{31}^{\left(l\right)}+e_{32}^{\left(l\right)}+\left(l+2\right)e_{33}^{\left(l\right)}\right)U^{\prime }+ikb\left(e_{32}^{\left(l\right)}+e_{24}^{\left(l\right)}\right)W^{\prime }-\left(l+2\right)\in_{33}^{\left(l\right)}Y^{\prime }]+\\ r^{l}[\left(\left(l+1\right)\left(e_{31}^{\left(l\right)}+e_{32}^{\left(l\right)}\right)-k^{2}b^{2}e_{24}^{\left(l\right)}\right)U+ikb\left(\left(l+1\right)e_{32}^{\left(l\right)}-e_{24}^{\left(l\right)}\right)W+k^{2}b^{2}\in_{22}^{\left(l\right)}Y]\}X\left(r\right)+\\ \left(\delta (r-a)-\delta (r-b)\right)\frac{1}{h^{l}}\{r^{l+2}\left(e_{33}^{\left(l\right)}U^{\prime }-\in_{33}^{\left(l\right)}Y^{\prime }\right)+r^{l+1}\left[\left(e_{31}^{\left(l\right)}+e_{32}^{\left(l\right)}\right)U+ikbe_{32}^{\left(l\right)}W\right]\}=0 \end{array}$$

where *U*, *V*, and *W* represent the amplitude of vibration in the *r*, *θ*, and *φ* directions. The prime denotes the derivative with respect to *r*.

It is obvious that Equation (7b) is independent, which represents the SH wave. The other three equations are coupled with each other, which are associated to the Lamb-like wave. Equation (7b) is relatively easy to be solved analytically, and it has obtained much attention, so here we just consider the solution of the Lamb-like wave.

We expand the field quantities into the Legendre polynomial basis:

(8) $$U(r)=\sum_{m=0}^{\infty }p_{m}^{1}Q_{m}(r),\;V(r)=\sum_{m=0}^{\infty }p_{m}^{2}Q_{m}(r),Y(r)=\sum_{m=0}^{\infty }p_{m}^{3}Q_{m}(r)\;$$

where $p_{m}^{\alpha }(\alpha =1,2,3)$ are the expansion coefficients, $Q_{m}(r)$ is an orthonormal set of polynomials in interval [*a*, *b*], and $Q_{m}(r)=\sqrt{\frac{2m+1}{h}}P_{m}(\frac{2r-(b+a)}{h})$, with $P_{m}$ being the polynomial of order *m*.

Substituting Equation (8) into Equations (7a), (7c), and (7d), then multiplying both sides of each modified equation by a complex conjugate $Q_{{}_{j}}^{*}(r)$ with *j* from 0 to *M*, integrating over *r* in the interval [*a*, *b*], reorganizing these equations into a matrix form and letting *k* become more apparent, we can get:

(9) $$k^{2}A\cdot p+k^{1}B\cdot p+C\cdot p=-\omega^{2}H\cdot p\;$$

where **A**, **B**, **C**, and **H** are matrices of the order 3(*M* + 1)·3(*M* + 1), which can be obtained by Equation (7), and is given in the Appendix $p={\left[p_{m}^{1}\;p_{m}^{2}\;p_{m}^{3}\right]}^{T}$.

Equation (9) is a positive-definite eigenvalue problem with real roots *ω*^2^. Note that in previous research work (see [10,11]), Equation (9) was transformed into an eigenvalue problem with eigenvalue *ω.* For the propagating wave, it is very efficient to specify real *k*, and then solve for *ω*. But for a non-propagating wave, the previous approach is useless because *k* is complex. It involves a multivariable search, including the search of the real and imaginary parts of *k* for a given real *ω*. In order to overcome this difficulty, we make some improvements regarding the solving process, which must be done in order to obtain the complete solutions, including the real, imaginary, and complex wave number solutions.

Introducing a new vector q=k⋅p, then substituting it into Equation (9), leads to:

(10) k⋅A⋅q+B⋅q=−(C−H)p 

Multiplying two sides of Equation (10) by inverse matrix **A**^−1^, and rearranging the terms yields:

(11) $$A^{-1}(H-C)p-(A^{-1}B)q=k\cdot q\;$$

Combining Equation (11) and the above vector q=k⋅p, and assuming $R={\left[p\;q\right]}^{T}$, we have:

(12) $$\left[\begin{array}{cc} 0 & I\\ A^{-1}\left(H-C\right) & -A^{-1}B \end{array}\right]R=k\cdot R\;$$

where **I** is the identity matrix.

The problem is then transformed into an eigenvalue problem with complex eigenvalues *k*(*ω*), which can be solved using the routine “Eigenvalues” function of Mathematica.

The computation technique for the electricity closed circuit case is similar to the electricity open-circuit case. For the traction-free and electricity closed circuit boundary conditions, we need to modify Equations (2) and (8) as follows:

(13) $$T_{ij}=\left(C_{kl}\varepsilon_{ij}-e_{kl}E_{ij}\right)X(z),\;D_{i}=e_{kl}\varepsilon_{ij}+\in_{kl}E_{i}\;$$

(14) $$U(r)=\sum_{m=0}^{\infty }p_{m}^{1}Q_{m}(r),V(r)=\sum_{m=0}^{\infty }p_{m}^{2}Q_{m}(r),Y(r)=\sum_{m=0}^{\infty }p_{m}^{3}Q_{m}(r)(r-a)(r-b)\;$$

Similarly, we can obtain the following governing the differential equations:

(15a) $$\begin{array}{l} \frac{1}{h^{l}}\{r^{l+2}\left(C_{33}^{\left(l\right)}U^{\prime \prime }+e_{33}^{\left(l\right)}\left(Y^{\prime \prime }(r-a)(r-b)+2Y^{\prime }(2r-a-b)+2Y\right)\right)+r^{l+1}[\left(l+2\right)C_{33}^{\left(l\right)}U^{\prime }+ikb\left(C_{23}^{\left(l\right)}+C_{44}^{\left(l\right)}\right)W^{\prime }+\\ (\left(l+2\right)e_{33}^{\left(l\right)}-e_{31}^{\left(l\right)}-e_{32}^{\left(l\right)})\left(Y^{\prime }(r-a)(r-b)+Y(2r-a-b)\right)]+r^{l}[\left(l+1\right)\left(C_{13}^{\left(l\right)}+C_{23}^{\left(l\right)}\right)U-\left(C_{11}^{\left(l\right)}+C_{22}^{\left(l\right)}+2C_{12}^{\left(l\right)}+k^{2}b^{2}C_{44}^{\left(l\right)}\right)U\\ +ikb(\left(l+1\right)C_{23}^{\left(l\right)}-C_{12}^{\left(l\right)}-C_{22}^{\left(l\right)}-C_{44}^{\left(l\right)})W-k^{2}b^{2}e_{24}^{\left(l\right)}Y(r-a)(r-b)]\}X\left(r\right)+\left(\delta (r-a)-\delta (r-b)\right)\frac{1}{h^{l}}\{r^{l+2}(C_{33}^{\left(l\right)}U^{\prime }\\ +e_{33}^{\left(l\right)}\left(Y^{\prime }(r-a)(r-b)+Y(2r-a-b)\right))+r^{l+1}\left[\left(C_{13}^{\left(l\right)}+C_{23}^{\left(l\right)}\right)U+ikbC_{23}^{\left(l\right)}W\right]\}=-\frac{\rho^{\left(l\right)}r^{l+2}\omega^{2}}{h^{l}}UX\left(r\right) \end{array}$$

(15b) $$\begin{array}{l} \frac{1}{h^{l}}\{r^{l+2}C_{44}^{\left(l\right)}W^{\prime \prime }+r^{l+1}[\left(ikbC_{23}^{\left(l\right)}+ikbC_{44}^{\left(l\right)}\right)U^{\prime }+\left(l+2\right)C_{44}^{\left(l\right)}W^{\prime }+ikb\left(e_{32}^{\left(l\right)}+e_{24}^{\left(l\right)}\right)(Y^{\prime }(r-a)(r-b)+Y(2r-a-b))]\\ +r^{l}[ikb\left(\left(l+2\right)C_{44}^{\left(l\right)}+C_{12}^{\left(l\right)}+C_{22}^{\left(l\right)}\right)U+\left(C_{66}^{\left(l\right)}-\left(l+2\right)C_{44}^{\left(l\right)}-k^{2}b^{2}C_{22}^{\left(l\right)}\right)W+ikb\left(l+2\right)e_{24}^{\left(l\right)}Y(r-a)(r-b)]\}X\left(r\right)\\ +\left(\delta (r-a)-\delta (r-b)\right)\frac{1}{h^{l}}\{r^{l+2}C_{44}^{\left(l\right)}W^{\prime }+r^{l+1}(ikbC_{44}^{\left(l\right)}U-C_{44}^{\left(l\right)}W+ikbe_{24}^{\left(l\right)}Y(r-a)(r-b))\}=-\frac{\rho^{\left(l\right)}r^{l+2}\omega^{2}}{h^{l}}WX\left(r\right) \end{array}$$

(15c) $$\begin{array}{l} \frac{1}{h^{l}}\{r^{l+2}\left(e_{33}^{\left(l\right)}U^{\prime \prime }-\in_{33}^{\left(l\right)}\left(Y^{\prime \prime }(r-a)(r-b)+2Y^{\prime }(2r-a-b)+2Y\right)\right)+r^{l+1}[(e_{31}^{\left(l\right)}+e_{32}^{\left(l\right)}+\left(l+2\right)e_{33}^{\left(l\right)})U^{\prime }\\ +ikb\left(e_{32}^{\left(l\right)}+e_{24}^{\left(l\right)}\right)W^{\prime }-\left(l+2\right)\in_{33}^{\left(l\right)}\left(Y^{\prime }(r-a)(r-b)+Y(2r-a-b)\right)]+r^{l}[\left(\left(l+1\right)\left(e_{31}^{\left(l\right)}+e_{32}^{\left(l\right)}\right)-k^{2}b^{2}e_{24}^{\left(l\right)}\right)U\\ +ikb\left(\left(l+1\right)e_{32}^{\left(l\right)}-e_{24}^{\left(l\right)}\right)W+k^{2}b^{2}\in_{22}^{\left(l\right)}Y(r-a)(r-b)]\}=0 \end{array}$$

The rest of the deduction process is similar, and the other boundary cases are not given here, to save space.

## 3. Numerical Results

In the paper, the FGPM spherical curved plate is composed of PZT-4 (inner surface) and Ba_2_NaNb_5_O_15_ (outer surface). Their material parameters are given in Table 1. We use the Voigt-type model, as described in the literature [12], to calculate the effective material property, which is expressed as:

(16) $$F(r)=F_{P}+(F_{B}-F_{P})V_{B}(r)\;$$

where *F_P_* and *F_B_* respectively represent the material properties of PZT-4 and Ba_2_NaNb_5_O_15_. *V_B_* represents the volume fraction of Ba_2_NaNb_5_O_15_. In this work, we consider four different gradient fields, $V_{B}(r)={\left(\frac{r-a}{h}\right)}^{n}$, *n* = 1, 2, and 3, namely the linear, quadratic, and cubic graded fields, and the sinusoidal graded field $V_{B}(r)=\sin(\frac{\pi }{2}\frac{r-a}{h})$.

### 3.1. Approach Validation and Convergence of the Problem

**Figure 2:**
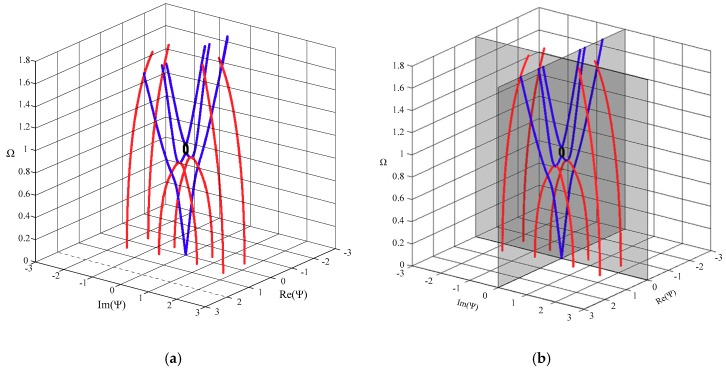
Dispersion curves of first three Lamb modes for a steel plate; real branch in blue, purely imaginary branch in black, complex branch in red. (**a**) Authors’ results, (**b**) Quintanilla et al.’s results from the spectral collocation method.

Since there has been no research on the non-propagating waves in FGPM structures before, we compute an isotropic plate, and we compare our results with the known results in the literature [20] from the spectral collocation method to check the validity of our approach. The resulting dispersion curves for a spherical curved steel plate with a big radius-thickness ratio (*η* = 100, *h* = 1 mm), approximately regarded as a flat plate, are shown in Figure 2. The material parameters are *ρ* = 7932 kg/m^3^, *C*_11_ = 281.757 GPa, *C*_12_ = 113.161 GPa, and *C*_44_ = 84.298 GPa, and the others are zero. For the three-dimensional plot, we adopt the conventional non-dimensional axis convention. The non-dimensional wave number and frequency are defined by Ψ=kh/π and $\Omega =\frac{\omega h}{\pi }\sqrt{\frac{\rho }{C44}}$. It clearly shows that the results obtained by the present approach agree well with the available results from the spectral collocation method.

**Figure 3:**
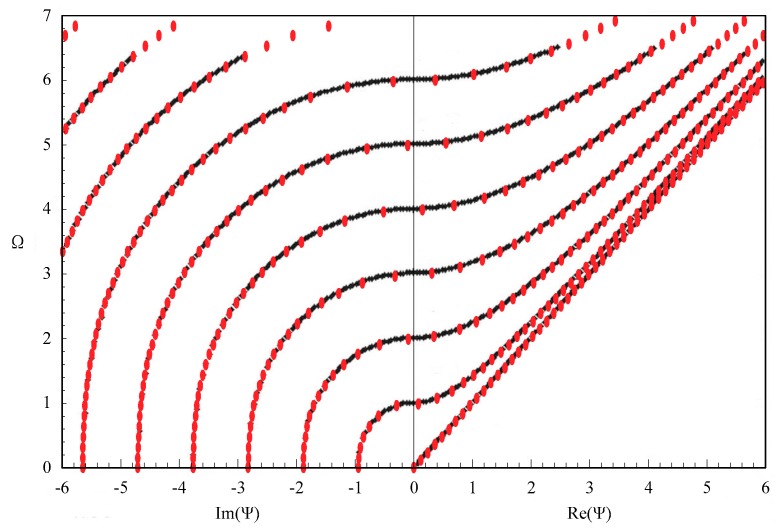
Dispersion curves of the shear horizontal (SH) wave in a piezoelectric plate; red dotted lines—our results, black ones—literature results.

The material in the above example is isotropic. We then also calculate the dispersion curves of SH wave in a piezoelectric plate, and make a comparison with the available results in the literature [21], which serves as a further validation of our approach. The FGPM degenerates to a homogeneous, piezoelectric material. The material is GaAs, and the parameters given in the literature [21] are *C*_11_ = *C*_22_ = *C*_33_ = 118.8 GPa, *C*_44_ = *C*_55_ = *C*_66_ = 59.4 GPa, *C*_12_ = *C*_13_ = 53.8 GPa, *e*_14_ = *e*_25_ = *e*_36_ = 0.154 C/m, ϵ_11_ = ϵ_22_ = ϵ_33_ = 110.625e^−12^ F/m^2^, *ρ* = 5307 kg/m^3^, and *h* = 1 mm. The resulting dispersion curves of the SH wave are shown in Figure 3, where the black dotted lines are the analytical results in the literature, and the red ones are our results. The numerical solutions by using our approach are entirely consistent with the analytical results, which show that the present approach is an effective method in solving guided wave problems.

**Figure 4:**
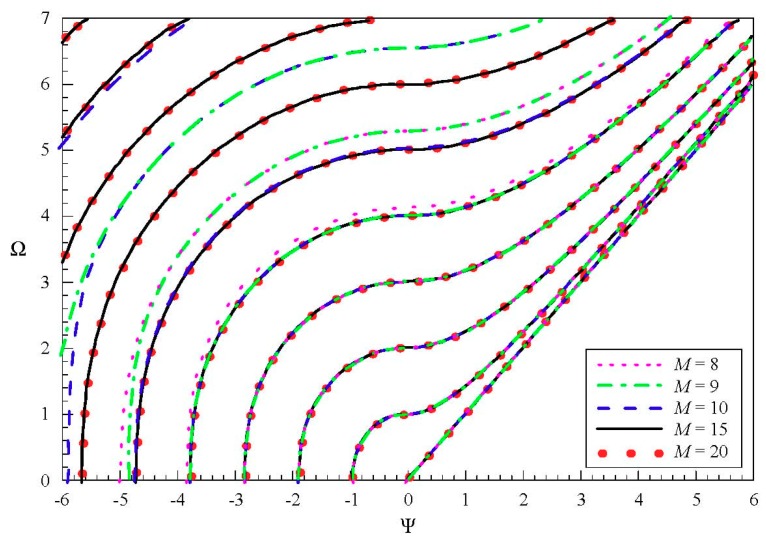
Dispersion curves of the SH wave in a piezoelectric plate for various “*M*”.

We then discuss the convergence of the present approach. We calculate the dispersion curves of SH wave in the above GaAs plate, for various “*M*”, as shown in Figure 4. It can be seen that more and more order modes converge as *M* increases. When *M* = 8, the first four modes are convergent. The first five converge when *M* = 9, the first six when *M* = 10, and the first ten when *M* = 15. Thus, we can think that at least the first *M*/2 modes are convergent. From these results, a good convergence of the present approach can be observed. Similarly, this can be concluded for the Lamb-like wave. In order to save space, it is not shown here. We take *M* = 30 in this paper.

### 3.2. Complete Frequency Spectrum for a FGPM Spherical Curved Plate

**Figure 5:**
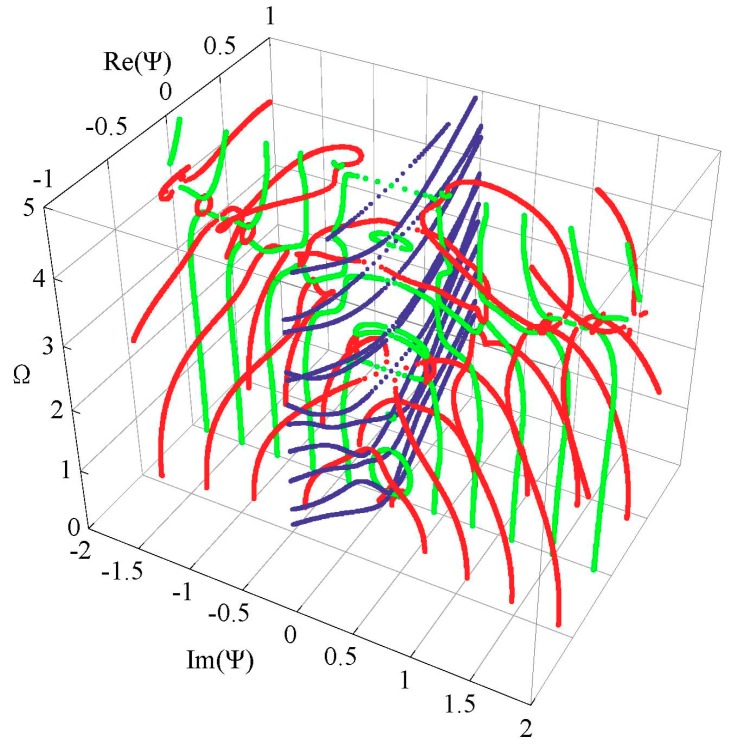
Complete frequency spectrum of a Lamb-like wave in a linear functionally graded piezoelectric material (FGPM) spherical curved plate; real branch in blue, purely imaginary branch in green, complex branch in red.

**Figure 6:**
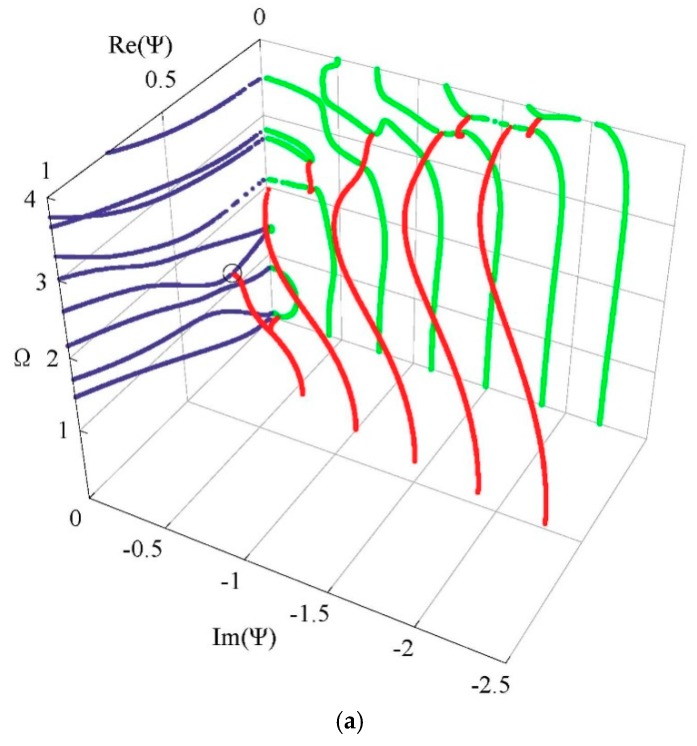

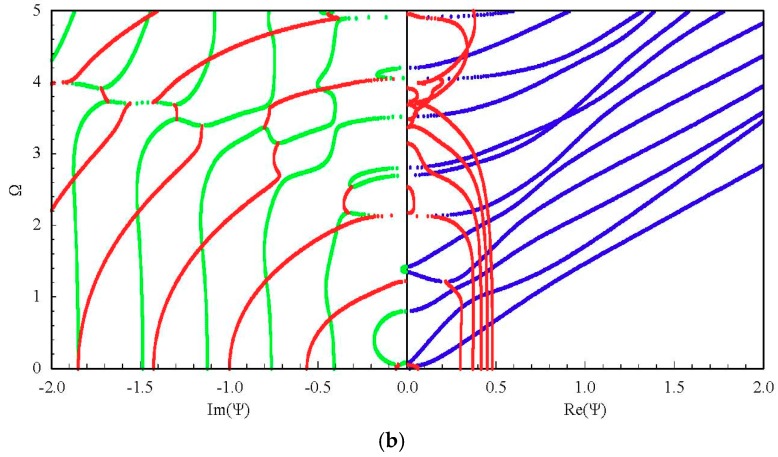
Frequency spectrum of a Lamb-like wave: (**a**) first quadrant curves; (**b**) 2D projection curves.

Generally, 3D dispersion curves can provide a clearer visualization and a more in-depth understanding of the characteristics for the guided wave. Figure 5 shows the complete 3D frequency spectrum of the Lamb-like wave in a linear FGPM spherical curved plate with an open circuit, *η* = 10 and *a* = 9 mm. From these curves, it can be clearly seen that there exists a finite number of real wave modes, and an infinite number of imaginary and complex wave modes, at any given frequency. The purely real and imaginary branches always arise in pairs of opposite signs. The complex ones arise in quadruples of complex conjugates. Purely imaginary and complex solutions correspond to the non-propagating wave. In view of the symmetry, Figure 6a shows one quadrant of the spectrum, and Figure 6b shows the projection onto the Ω-Re(Ψ) and Ω-Im(Ψ) planes for clarity. For purely imaginary branches, most begin at the Ω = 0 plane, and they terminate at cut-off frequencies on the Ω axis, while a few with small wave numbers begin a certain cut-off frequency and end at the adjacent one. For complex branches, most begin at the Ω = 0 plane and terminate at the local minima of the other branches, at low frequency. The real parts of these modes are usually small, and they reduce with increasing frequency, while a few interlinking the gap between two neighboring imaginary branches at high frequency appear.

**Figure 7:**
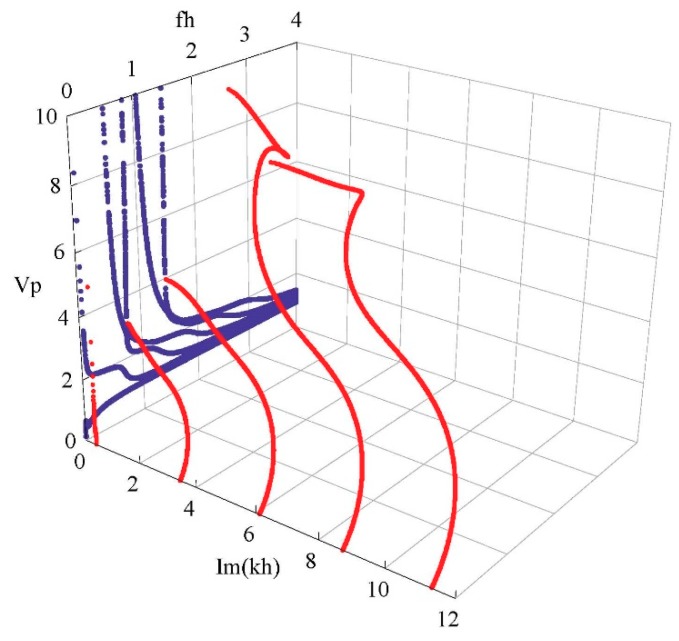
Phase velocity dispersion and attenuation curves of a Lamb-like wave; propagating wave in blue; non-propagating wave in red.

Figure 7 shows the phase velocity dispersion and attenuation curves of the first few real and complex branches. The dimensionless phase velocity and frequency and wave number are defined by Vp=ω/(Re(k)⋅C55/ρ), $fh=\omega h/(2\pi \sqrt{C_{55}/\rho })$, and Im(kh), respectively. Apparently, with increasing frequency, the phase velocity of a propagating mode gradually decreases to a steady value, but the velocity of an non-propagating mode increases, and is far greater than that of a propagating mode. For instance, the phase velocity of the fifth complex branch is beyond 6 at *fh* = 3–4, but that of the real branches is below 2. Moreover, the wave dispersion is quite weak in this frequency range.

### 3.3. Influences of Piezoelectricity and Boundary Conditions on Frequency Spectrum

**Figure 8:**
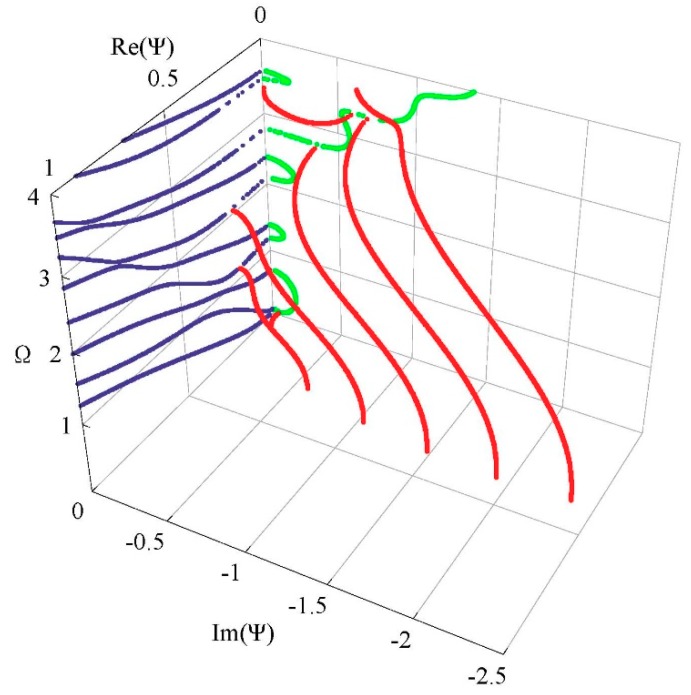
Frequency spectrum of a Lamb-like wave in a FGM spherical curved plate.

To investigate the influences of material piezoelectricity on the frequency spectrum, we assume the piezoelectric and dielectric coefficients are zero while the others are invariant. Then, the FGPM spherical curved plate degenerates to a FGM one. The resulting frequency spectrum of the FGM spherical curved plate is shown in Figure 8. Comparison with Figure 6a, the remarkable influences of piezoelectricity on the propagating and non-propagating wave can be found. The influence on the non-propagating wave is more significant. Those imaginary branches beginning at Ω = 0 plane and terminating at cut-off frequencies disappear in the FGM spherical curved plate. The complex branches are also very different, and another local inflection point appears on the sixth real branches as the frequency increases. As suggested by Onoe et al. [29], the complex branch is sensitive to material properties.

**Figure 9:**
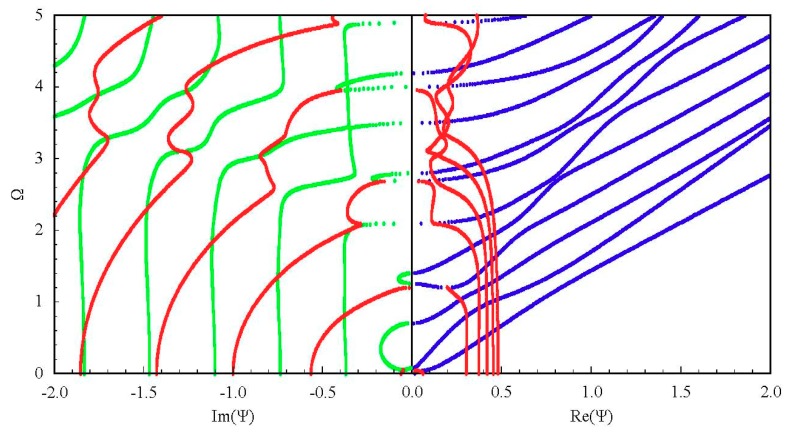
Frequency spectrum of a Lamb-like wave for mechanical free and electricity closed-circuit boundary conditions.

**Figure 10:**
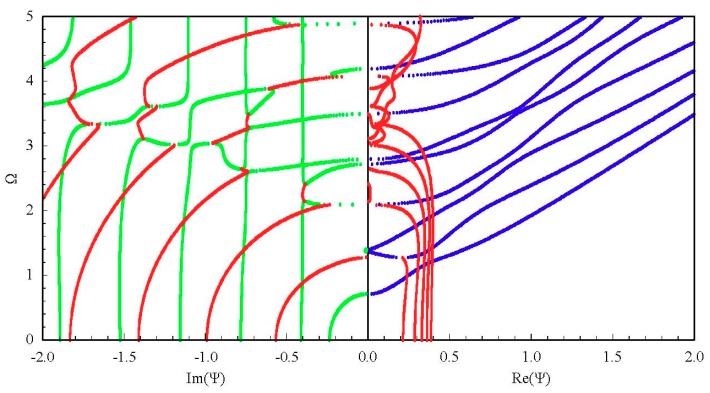
Frequency spectrum of a Lamb-like wave for mechanical fixed boundary and electricity closed-circuit boundary conditions.

Figure 9 and Figure 10 present the obtained frequency spectrum of the FGPM spherical curved plates with different mechanical and electrical boundary conditions. Comparing Figure 9 with Figure 6b, we can notice that electricity boundary condition has a more significant effect on the complex branches than that on the real branches. The complex branches connecting two imaginary branches disappear. A comparison of Figure 6b with Figure 10 indicates that the effect of mechanical boundary condition on lower order modes is greater than that on higher order modes. There are two propagating modes below the first cut-off frequency for the mechanical free boundary condition, but these two modes do not exist under the mechanical fixed boundary condition.

### 3.4. Influences of Graded Field on the Frequency Spectrum

**Figure 11:**
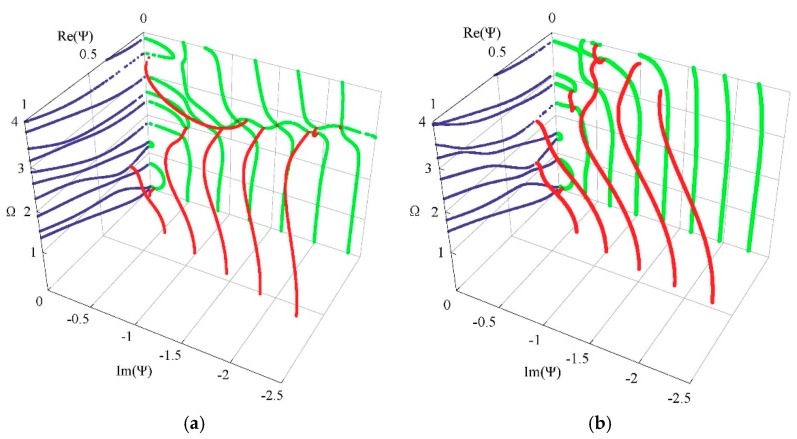
Frequency spectrum: (**a**) cubic graded field, (**b**) sinusoidal graded field.

**Figure 12:**
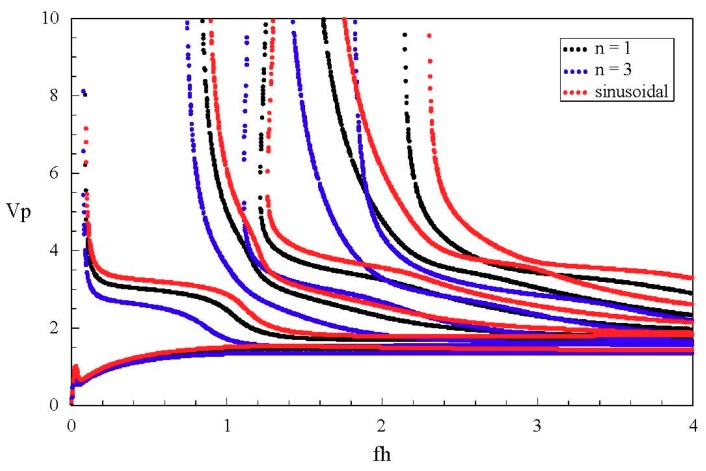
Phase velocity dispersion curves of the Lamb-like wave.

**Figure 13:**
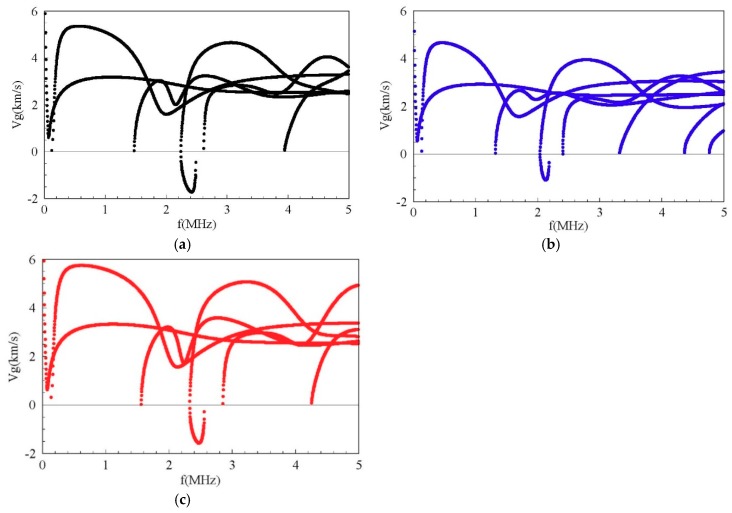
Group velocity dispersion curves of the Lamb-like wave for different FGMP spherical curved plates: (**a**) linear graded field, (**b**) cubic graded field, (**c**) sinusoidal graded field.

Considering the other two graded fields, cubic and sinusoidal graded shapes. The corresponding frequency spectra are given in Figure 11. The results show that the graded fields have significant influences on the dispersion characteristics. From a comparison between Figure 11 and Figure 6a, we can notice that the imaginary part of the complex branches for the sinusoidal graded case, at the Ω = 0 plane, it is bigger than that for the other two cases. Interestingly, for the sinusoidal graded cases, there exists an inflection point on the sixth real branch. For clarity, we also calculate the phase velocity and group velocity dispersion curves of the Lamb-like propagating wave for the three graded fields, as shown in Figure 12 and Figure 13. The phase velocity and group velocity for the sinusoidal case is bigger than that for the linear one, while the linear case is bigger than the cubic case. That is because different graded fields lead to different material volume distributions. The Ba_2_NaNb_5_O_15_ content for the sinusoidal graded field is the highest, and the wave velocity of Ba_2_NaNb_5_O_15_ is bigger than that of PZT-4.

### 3.5. Displacement and Electric Potential Fields

**Figure 14:**
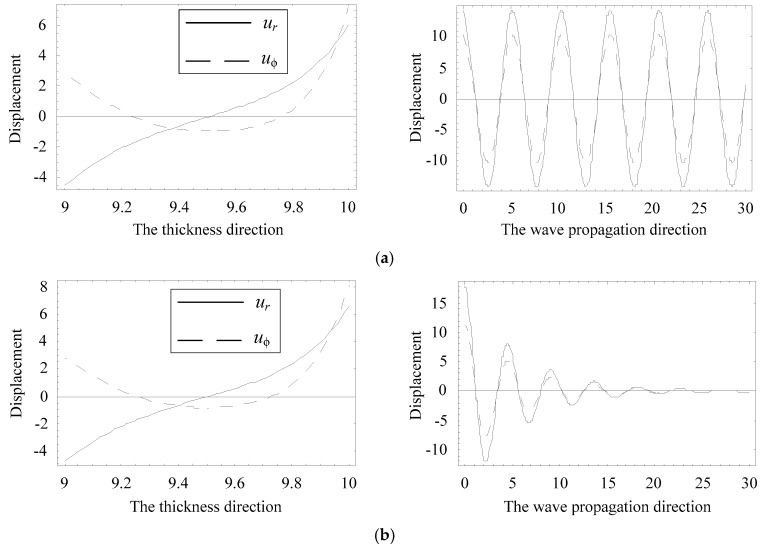
Distributions of the displacement: (**a**) Ω = 1.21815, Ψ = 0.19264, (**b**) Ω = 1.21019, Ψ = 0.21937–0.02803i.

**Figure 15:**
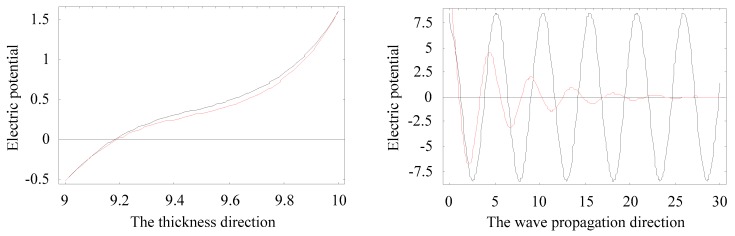
Distributions of the electric potential: black lines for Ω = 1.21815, Ψ = 0.19264, red lines for Ω = 1.21019, Ψ = 0.21937–0.02803i.

The amplitude distributions of physical quantities, displacement, and electric potential fields, can be obtained according to Equations (6) and (8). We select a special position where the real branch firstly occurs an inflection point, at about Ω = 1.2, as marked with a circle in Figure 6a. Figure 14 and Figure 15 present the distributions of the physical quantities in the *r* direction and wave propagating direction, when Ω = 1.21019, Ψ = 0.21937–0.02803 i, and Ω = 1.21815, Ψ = 0.19264, respectively. These figures reveal that the complex branch exhibits an oscillatory distribution and propagates a very long distance (about a few tenths of the thickness). The real branch propagates without any attenuation. The displacement *u_r_* and electric potential distributions change along the thickness direction in a nearly anti-symmetric manner. Moreover, the displacements of *u_r_* are very similar at the two frequencies, implying that the non-propagating wave mode will turn into a propagating wave mode with increasing frequency.

## 4. Conclusions

This paper presents a method based on the orthogonal function technique to compute the complete 3D spectrum, including real, purely imaginary, and complex branches, for guided wave problems in FGPM spherical curved plates. A good agreement between our results and available numerical ones confirms the correctness of our approach. The method throws new light onto guided wave problems involving composite materials or curved structures, which are usually very demanding for traditional methods. Characteristics of non-propagating Lamb-like waves in FGPM spherical curved plates are investigated. Based on the above numerical results, some interesting conclusions can be drawn:

(1) The presented method can transform the set of differential wave equations into an eigenvalue problem, thus obtaining the complete solution straightforwardly, which avoids the iterative search procedure of the traditional methods to find the complex roots;
(2) Some complex branches of the Lamb-like waves can propagate a quite long distance (more than 10 times the plate thickness). These modes will turn into the propagating modes with increasing frequency. Complex non-propagating modes exhibit both local vibration and local propagation, and purely imaginary non-propagating modes exhibit only local vibration and no local propagation;
(3) Some non-propagating modes have a noticeably higher phase velocity than the propagating modes. Also, the wave dispersion of the non-propagating mode is quite weak in a certain frequency range;
(4) The piezoelectricity, graded field, and mechanical and electrical boundary conditions have significant influences on non-propagating waves.

## Figures and Tables

**Table 1:** Material parameters of two piezoelectric materials.

**Property**	***C*_11_**	***C*_12_**	***C*_13_**	***C*_22_**	***C*_23_**	***C*_33_**	***C*_44_**	***C*_55_**	***C*_66_**
Ba_2_NaNb_5_O_15_	23.9	10.4	5.0	24.7	5.2	13.5	6.5	6.6	7.6
PZT-4	13.9	7.8	7.4	13.9	7.4	11.5	2.56	2.56	3.05
	***e*** **_15_**	***e*** **_24_**	***e*** **_31_**	***e*** **_32_**	***e*** **_33_**	**ϵ** **_11_**	**ϵ** **_22_**	**ϵ** **_33_**	***ρ***
Ba_2_NaNb_5_O_15_	2.8	3.4	−0.4	−0.3	4.3	196	201	28	5.3
PZT-4	12.7	12.7	−5.2	−5.2	15.1	650	650	560	7.5

Units. *C_ij_* (10^10^ N/m^2^), ϵ*_ij_* (10^−11^ F/m^2^), *e_ij_* (C/m), *ρ* (10^3^ kg/m^3^).

## Appendix A

The explicit expressions for the elements are:

$$\begin{array}{l} {}^{l}A_{11}^{j,m}=-\frac{1}{h^{l}}b^{2}C_{44}^{\left(l\right)}\beta (m,l,0,j),{}^{l}A_{13}^{j,m}=-\frac{1}{h^{l}}b^{2}e_{24}^{\left(l\right)}\beta (m,l,0,j),{}^{l}A_{22}^{j,m}=-\frac{1}{h^{l}}b^{2}C_{22}^{\left(l\right)}\beta (m,l,0,j),{}^{l}A_{31}^{j,m}=-\frac{1}{h^{l}}b^{2}e_{24}^{\left(l\right)}\beta (m,l,0,j),\\ {}^{l}A_{33}^{j,m}=\frac{1}{h^{l}}b^{2}\in_{22}^{\left(l\right)}\beta (m,l,0,j),{}^{l}A_{12}^{j,m}={}^{l}A_{21}^{j,m}=0,\;{}^{l}A_{23}^{j,m}={}^{l}A_{32}^{j,m}=0;\\ {}^{l}B_{12}^{j,m}=\frac{1}{h^{l}}\{ib\left(C_{23}^{\left(l\right)}+C_{44}^{\left(l\right)}\right)\beta (m,l+1,1,j)+ib\left[\left(l+1\right)C_{23}^{\left(l\right)}-C_{12}^{\left(l\right)}-C_{22}^{\left(l\right)}-C_{44}^{\left(l\right)}\right]\beta (m,l,0,j)+ibC_{23}^{\left(l\right)}\gamma (m,l+1,0,j)\},\\ \;{}^{l}B_{21}^{j,m}=\frac{1}{h^{l}}\{ib\left(C_{23}^{\left(l\right)}+C_{44}^{\left(l\right)}\right)\beta (m,l+1,1,j)+ib[\left(l+2\right)C_{44}^{\left(l\right)}+C_{12}^{\left(l\right)}+C_{22}^{\left(l\right)}]\beta (m,l,0,j)+ibC_{44}^{\left(l\right)}\gamma (m,l+1,0,j)\},\\ {}^{l}B_{23}^{j,m}=\frac{1}{h^{l}}\{ib\left(e_{32}^{\left(l\right)}+e_{24}^{\left(l\right)}\right)\beta (m,l+1,1,j)+\left(l+2\right)ibe_{24}^{\left(l\right)}\beta (m,l,0,j)+ibe_{24}^{\left(l\right)}\gamma (m,l+1,0,j)\},\\ \;{}^{l}B_{32}^{j,m}=\frac{1}{h^{l}}\{ib\left(e_{32}^{\left(l\right)}+e_{24}^{\left(l\right)}\right)\beta (m,l+1,1,j)+ib\left[\left(l+1\right)e_{32}^{\left(l\right)}-e_{24}^{\left(l\right)}\right]\beta (m,l,0,j)+ibe_{32}^{\left(l\right)}\gamma (m,l+1,0,j)\},\\ {}^{l}B_{11}^{j,m}={}^{l}B_{22}^{j,m}={}^{l}B_{33}^{j,m}=0,{}^{l}B_{13}^{j,m}={}^{l}B_{31}^{j,m}=0;\\ \begin{array}{l} {}^{l}C_{11}^{j,m}=\frac{1}{h^{l}}\{C_{33}^{\left(l\right)}\beta (m,l+2,2,j)+\left(l+2\right)C_{33}^{\left(l\right)}\beta (m,l+1,1,j)+\left(l+1\right)\left(C_{13}^{\left(l\right)}+C_{23}^{\left(l\right)}\right)\beta (m,l,0,j)-\\ \;\left(C_{11}^{\left(l\right)}+C_{22}^{\left(l\right)}+2C_{12}^{\left(l\right)}\right)\beta (m,l,0,j)+C_{33}^{\left(l\right)}\gamma (m,l+2,1,j)+\left(C_{13}^{\left(l\right)}+C_{23}^{\left(l\right)}\right)\gamma (m,l+1,0,j)\} \end{array},\;\\ {}^{l}C_{13}^{j,m}=\frac{1}{h^{l}}\{e_{33}^{\left(l\right)}\beta (m,l+2,2,j)+\left[\left(l+2\right)e_{33}^{\left(l\right)}-e_{31}^{\left(l\right)}-e_{32}^{\left(l\right)}\right]\beta (m,l+1,1,j)+e_{33}^{\left(l\right)}\gamma (m,l+2,1,j)\},\;\\ \begin{array}{l} {}^{l}C_{22}^{j,m}=\frac{1}{h^{l}}\{C_{44}^{\left(l\right)}\beta (m,l+2,2,j)+\left(l+2\right)C_{44}^{\left(l\right)}\beta (m,l+1,1,j)+\left[C_{66}^{\left(l\right)}-\left(l+2\right)C_{44}^{\left(l\right)}\right]\beta (m,l,0,j)+\\ \;C_{44}^{\left(l\right)}\gamma (m,l+2,1,j)-C_{44}^{\left(l\right)}\gamma (m,l+1,0,j)\} \end{array},\;\\ \begin{array}{l} {}^{l}C_{31}^{j,m}=\frac{1}{h^{l}}\{e_{33}^{\left(l\right)}\beta (m,l+2,2,j)+\left[e_{31}^{\left(l\right)}+e_{32}^{\left(l\right)}+\left(l+2\right)e_{33}^{\left(l\right)}\right]\beta (m,l+1,1,j)+\left(l+1\right)\left(e_{31}^{\left(l\right)}+e_{32}^{\left(l\right)}\right)\beta (m,l,0,j)+\\ \;e_{33}^{\left(l\right)}\gamma (m,l+2,1,j)+\left(e_{31}^{\left(l\right)}+e_{32}^{\left(l\right)}\right)\gamma (m,l+1,0,j)\} \end{array},\;\\ {}^{l}C_{33}^{j,m}=\frac{1}{h^{l}}\{-\in_{33}^{\left(l\right)}\beta (m,l+2,2,j)-\left(l+2\right)\in_{33}^{\left(l\right)}\beta (m,l+1,1,j)-\in_{33}^{\left(l\right)}\gamma (m,l+2,1,j)\},\\ {}^{l}C_{12}^{j,m}={}^{l}C_{21}^{j,m}=0,\;{}^{l}C_{23}^{j,m}={}^{l}C_{32}^{j,m}=0;\\ {}^{l}H_{m}^{j}=\frac{1}{h^{l}}\rho^{\left(l\right)}\beta (m,l+2,0,j); \end{array}$$

With:

$$\beta (m,l,n,j)=\int_{a}^{b}Q_{j}^{*}(r)r^{l}\frac{\partial^{n}Q_{m}(r)}{\partial r^{n}}dr;\;\gamma (m,l,n,j)=\int_{a}^{b}Q_{j}^{*}(r)r^{l}\frac{\partial X(r)}{\partial r}\frac{\partial^{n}Q_{m}(r)}{\partial r^{n}}dr\;$$
